# Of oncogenes and open science: an interview with Harold Varmus

**DOI:** 10.1242/dmm.038919

**Published:** 2019-03-01

**Authors:** Harold Varmus

**Keywords:** Cancer, Oncogenes, Genetics, Disease

## Abstract

Harold Varmus has made pioneering contributions to our understanding of cancer as a genetic disease. The discovery of the cellular origin of retroviral oncogenes earned him and his long-term collaborator, Michael Bishop, the Lasker Prize for Basic Medical Sciences in 1982 and the Nobel Prize in Physiology and Medicine in 1989. Throughout his career, Varmus has held several leadership roles that shaped science policy in the US and worldwide, and he has been an outspoken advocate for open science. In this interview, he talks (among other things) about the factors that shaped his early career choices, the thrill of scientific discovery, and the importance of including diverse populations in genomic studies of cancer and other diseases.

**Introduction**

Harold Varmus is the Lewis Thomas University Professor and a research group leader at Weill Cornell Medicine and a Senior Associate Member of the New York Genome Center, where he is helping to coordinate Polyethnic 1000, an initiative to study cancer genomes in ethnically diverse patient populations. He started down his research path in 1968 in Ira Pastan's laboratory at the National Institutes of Health (NIH), where he investigated regulation of gene expression in bacteria. In 1970, he moved to the University of California San Francisco to study RNA tumor viruses and began his longstanding collaboration with Michael Bishop. Their discovery of the cellular proto-oncogene c-*src* as the precursor of the Rous Sarcoma Virus cancer gene, v-*src*, led to fundamental changes in our understanding of cancer. For this discovery, they were jointly awarded the Nobel Prize in Physiology and Medicine in 1989. Varmus has actively shaped science policy through his roles as Director of both the NIH and of the National Cancer Institute (NCI) and various other activities in the scientific enterprise.

**Figure:**
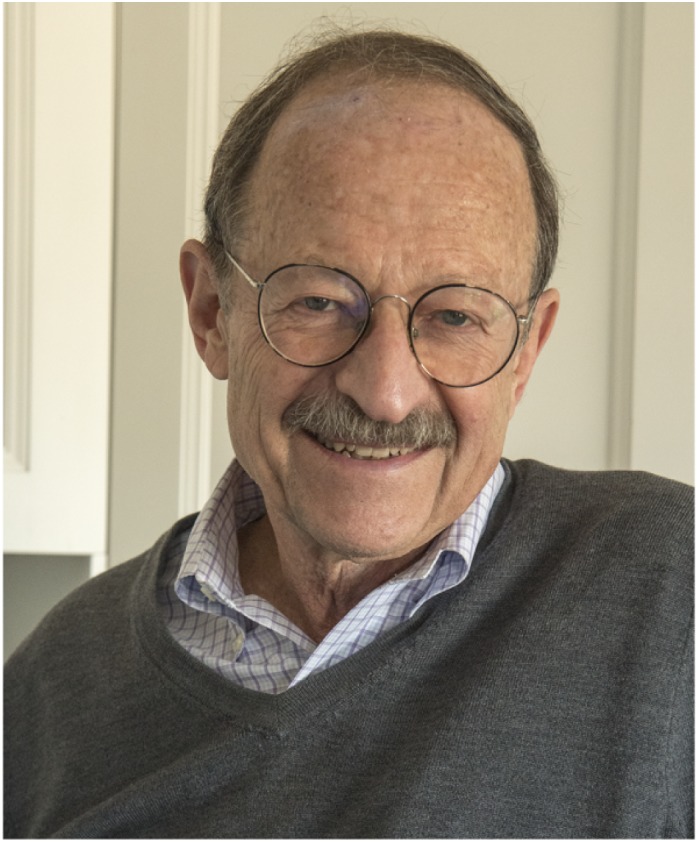
Reproduced with permission from Jon Friedman, who was commissioned by the National Cancer Institute. This image is not published under the terms of the CC-BY license of this article. For permission to reuse, please see http://jonrfriedman.com/.

**We usually ask our interviewees if they always wanted to be scientists. But your path towards a scientific career was a little unusual. Could you tell us about it?**

It was unusual. I was brought up with the assumption that I was going into medicine because my father was a doctor, which is a career fate that used to be viewed as hereditary, especially among Jewish males growing up in the New York area. But I always had a lot of other interests, and I was not fired up by science in high school as a lot of my current colleagues were. If someone had asked me then what I most cared about, it would have been novels and sports. When I got to college, I found that the pre-med courses were boring and that literature was exciting, and I was more interested in running the college newspaper and in talking about plays and poetry than about spending time in a laboratory.

I carried on with my pre-med courses and applied to medical school after graduation, but I also considered other things like travel fellowships and graduate programs in English. When I received a Woodrow Wilson Fellowship to study English literature, starting graduate school at Harvard seemed to be the right thing to do. A summer trip to Europe after graduation with my friend and Amherst classmate, Arthur Landy (now an Emeritus Professor of medical science at Brown), brought us to Moscow to the 1961 International Congress of Biochemistry, at which the genetic code was announced. He was incredibly excited about this, much more than I was about the things I was reading to prepare for my graduate work. The following year, my friends at Harvard Medical School seemed to be having a better time than I had in the English department. These things persuaded me to go to medical school in 1962, as I thought it would give me more options than a PhD in literature would.

I attended a medical school (Columbia's College of Physicians and Surgeons) that had frequent guest seminars from scientists who were giving talks about what was happening in molecular biology. I realized that a frontier was opening and that we were going to understand disease in unprecedented detail. Those were incentives that convinced me to pursue an academic path in medicine.

**Was witnessing those incredible advances in molecular biology the main motivation to pursue lab work?**

Well, it was a little more complex than that. While I was interested in how medicine was changing and becoming more molecular and genetic, I really wasn't thinking about myself at the bench. I saw myself as someone involved in the clinic and with students, practicing and teaching a more scientific form of medicine. I had spent a few summers in labs, but didn't find those experiences particularly satisfying. So the main driver that brought me to the bench was the Vietnam War. Everyone with an MD was obliged to serve the country. Most people joined the armed forces, but there was an option for those of us who vehemently opposed the conflict to work for one of the agencies of the US Public Health Service, such as the NIH. The NIH seemed to lead most clearly towards the kind of career I was interested in. I didn't get matched with any of the older and more famous NIH investigators, but I was lucky, as it turns out, to get into Ira Pastan's newly established lab. Ira was working mainly on hormone release by the thyroid gland but had just started to study regulation of *lac* gene expression by the universal messenger, cyclic AMP, in bacteria. Ira and I were learning molecular biology together; with him, I learned the thrill of running a novel experiment and getting an interesting result. The most important day of my life as a scientist was when I realized I actually had an assay that could accurately measure messenger RNA, a gauge of expression of a single gene. This may seem trivial today in the age of single-cell transcriptomics, but in 1968 it was thrilling. So I went from just being glad to be in Bethesda and not in Vietnam to knowing I had a powerful tool that could answer big questions about gene regulation.

**A fascinating area of your current work is splicing factor mutations. Can you tell us how you came into the field?**

I was struck by early evidence that prompted the idea that mutant splicing factor genes could be oncogenic. The process is still mysterious. Several groups are working on four key splicing factors [SF3B1, U2AF1, SRSF2 and ZRSR2] that are recurrently mutated in cancer, yet we still don't understand what the oncogenic mechanism is. It is an opportunity, but it is also frustrating to me personally because I can't see the key experiments that would help us go about solving this problem.

A key issue in my opinion is that, although mutations in these factors change the pattern of RNA splicing, without killing cells, the cells continue to require a wild-type copy of the mutant gene. So, in a way, these mutations confer both a loss of function and a gain of function. How this mechanistically leads to cancer, we don't know. It is even possible that the mechanism has nothing to do with the splicing aberrations. One interesting angle we are exploring is that mutations in some other genes could form synthetic lethal combinations with splicing factor mutations, and such dependencies might define specific targets for development of drugs to treat cancers with splicing factor mutations. Although this translational aspect is important, I am much more curious about the mechanisms by which these mutations contribute to neoplasia.

“Although [the] translational aspect [of splicing factor mutations] is important, I am much more curious about the mechanisms by which these mutations contribute to neoplasia.”

**Speaking of synthetic lethality: although it is a well-established idea in cancer, we haven't been successful in translating it into therapy**

Well, it is still ‘early days’ for finding and exploiting synthetic lethalities. Moreover, the combination of PARP inhibitors with mutations affecting DNA repair machinery is a version of synthetic lethality that already has shown promise in patients. Another way to proceed is illustrated by our work in exploring mutual exclusivity of driver mutations affecting two commonly mutated genes, *EGFR* and *KRAS*, in lung cancers. We have recently published work in *eLife* showing that the level of RAS signaling has to be subtly regulated. Since levels of activities of certain kinases, such as ERK, matter, both kinases and phosphatases could be targeted with drugs to produce a synthetically lethal effect. Although our scientific community knows a lot about the RAS proteins and the signaling pathways they trigger, we have been unsuccessful in delivering RAS therapeutics, so understanding the effects of excessive signaling on cell fitness may provide a new approach to control of cancer.

**As Director of the NCI, you presided over the completion of The Cancer Genome Atlas (TCGA). Aside from the scientific insight, what do you think are the key lessons we learnt from a project of such magnitude?**

There was some initial resistance to TCGA, as there commonly is to large-scale plans for expensive team science, but the project now has widespread support, as researchers continue to mine and learn from the publicly available datasets. No project of this size is ever perfect: for instance, we weren't able to include as many cancer types as I would have liked because obtaining useful samples of certain kinds of tumors was harder than we anticipated.

Despite these shortcomings, TCGA provides a model for future projects. The New York Genome Center, where I'm also actively involved these days, recently launched the Polyethnic 1000 project. We aim to take advantage of the incredibly diverse patient populations in New York and of recent technological improvements to correct some of the limitations of TCGA – in particular the fact that the vast majority of samples in TCGA come from individuals of European ancestry. We're interested in exploring patterns of somatic and germline mutations in cancer patients of Asian, African and South American descent. Although it will be hard for a single center in a single city to carry out a project of this magnitude, the idea is to perform targeted pilot projects and form alliances to harness the diversity of *Homo sapiens* and understand how cancer arises in individuals of diverse genetic backgrounds who may also be exposed to different environmental factors.

**So you think there is a need for a ‘TCGA 2.0’ database with diverse representation and more information?**

Nearly all scientific databases need improved versions. In the case of genomic databases of cancers, we are lacking detailed clinical information, especially about responses to therapy and long-term outcomes, and we usually lack information about non-coding regions of the genome and about the relationship of somatic mutations and outcomes to inherited gene variation. Several research consortia, like the GENIE project or the Polyethnic 1000 initiative here in New York, are trying to repair these deficiencies, both at local and national levels. I hope there will be support for the aggregation and integration of clinical and genomic data in an accessible and sensible way, so that anyone can analyze the data for legitimate purposes.

**Speaking of open and accessible science, what do you think are the key steps the community still needs to take towards reaching this goal?**

We are making good progress through public digital libraries, like PubMed Central; through the growing number of true open-access journals; through the expanding use of preprint servers; and through the commitments of funding agencies to the idea of making the work they fund immediately and fully accessible. But I also think one of the big obstacles to freeing up scientific information remains the way in which we continue to pay allegiance to the idea that the most important work is published in so-called ‘high-impact’ journals that continue to restrict access by imposing highly lucrative subscription fees. These journals continue to thrive, despite a kind of anti-social policy, because so many academic scientists evaluate each other's work and measure abilities and accomplishments based on where people have published. This practice of ceding judgments to journal editors, rather than retain it among working scientists, has become ever more prevalent and powerful since the 70s and 80s. It has also led to increasing levels of sloppiness and irresponsibility – both by considering someone a good scientist because he or she has published a number of papers in elite journals, without regard to the science in those papers, and by creating false incentives for scientists (especially trainees seeking jobs) to produce findings that meet some arbitrary standard for acceptance by such journals. Most of my academic colleagues in fields outside of biomedicine cannot imagine evaluating somebody for a job or for tenure, or for a prize, without reading their work in detail. We need to get away from false metrics and return to the task of looking at our colleagues' work closely. I realize the magnitude of this problem – there are many people and many papers – but we are not being asked to evaluate the whole world, we are typically asked to evaluate a single person or a small group. We should let the candidates themselves tell us what their most important contributions to science have been and then evaluate them by reading their most important papers, regardless of where they appeared.

“We need to get away from false metrics and return to the task of looking at our colleagues’ work closely.”

Once we achieve that, we'll eliminate a lot of distinctions between journals. Then the prejudices that have allowed some journals to refuse to support open access will eventually vanish. We as a scientific community can undermine the power of certain journals by demanding that information is shared. We have to eliminate the current situation in which the fate of an individual researcher and their trainees depends on publishing in certain journals. Changing the landscape of scientific publishing will be hard, because current business plans still work, and publishers, both commercial and society-based, are reluctant to abandon their large profits.

The only way by which we'll eventually get out of the current situation is by changing the formula dramatically. That means that we'll probably have to move to a world where the authors have full control – their work will be presented online together with expert reviews and perhaps accompanied by a new evaluation system in which members of the scientific community will provide qualitative and perhaps quantitative measures of the value of the paper. The current world of high- and low-impact journals will eventually dissolve, it's just taking a lot longer than I thought.

A few of us started Public Library of Science (PLoS) early in the new millennium not to publish journals, but to persuade journal publishers to provide their content to PubMed Central within 6 months of publication. But, because of their career-determining influence, the journals were able to resist even this modest step towards access, let alone full and immediate open-access policies. As a result, it took a directive from Congress to mandate that all NIH-funded research must be made available in PubMed Central within a year. We are up against powerful organizations, but fortunately, things are changing. Slowly, but they are changing.

**You've been in leadership roles for most of your career and many junior scientists look up to you. Do you have any pearls of wisdom to share?**

Oh, don't ask me this question [laughs]. General advice is generally not very useful, because everyone's situation is different and a cliché won't solve any individual person's problem. I don't have advice for everybody. I suppose that I could recommend that everyone find someone they can trust and ask that person for good advice.

**We talked about your interest in literature. Is there anything else you enjoy outside of your academic work?**

I enjoy many things outside of work, especially the cultural delights of New York – art, theatre, music, opera, film – but can't pin down a single favorite. Sometimes, my interest in the arts and sciences overlap. For some years, for instance, I've been intermittently performing a show, called Genes and Jazz, with my son Jacob. It was a combination of his jazz and an illustrated talk about my views on evolution, genes and cancer. I am also a lover of exercise, especially cycling and tennis, mixed with some sculling, hiking and swimming.

